# Supplementary material for: HTLV-2 Enhances CD8+ T Cell-Mediated HIV-1 Inhibition and Reduces HIV-1 Integrated Proviral Load in People Living with HIV-1
Source: Viruses. 2022 Nov 9;14(11):2472. doi: 10.3390/v14112472 (PMC9695633; doi:10.3390/v14112472)
Supplement: Supplementary file 1 [file viruses-14-02472-s001.zip › viruses-1973548-supplementary.pdf]

Table S1: Antibodies catalogue number

Miltenyi Biotec

130-113-217: CD4-peridinin chlorophyll protein complex (PerCP)

130-110-632: CD45RA phycoerythrin (PE)

130-120-600: CCR7-allophycocyanin (APC)

170-081-075: CD8-phycoerythrin (PE)-Vio770

Beckman Coulter

B00070: human leucocyte antigen (HLA)-DR-Krome Orange (KrO)

B92396: CD38-Pacific Blue (PB)

A94680: CD3-allophycocyanin (APC)-A750
